# Scope and costs of autorefraction and photoscreening for childhood amblyopia—a systematic narrative review in relation to the EUSCREEN project data

**DOI:** 10.1038/s41433-020-01261-8

**Published:** 2020-11-30

**Authors:** Anna M. Horwood, Helen J. Griffiths, Jill Carlton, Paolo Mazzone, Arinder Channa, Mandy Nordmann, Huibert J. Simonsz, on behalf of The EUSCREEN Foundation

**Affiliations:** 1 University of Reading, Reading, UK; 2 Royal Berkshire Hospital, Reading, UK; 3 University of Sheffield, Sheffield, UK; 4 Erasmus Medical Centre, Rotterdam, The Netherlands

**Keywords:** Risk factors, Epidemiology

## Abstract

**Background:**

Amblyopia screening can target reduced visual acuity (VA), its refractive risk factors, or both. VA testing is imprecise under 4 years of age, so automated risk-factor photoscreening appears an attractive option. This review considers photoscreening used in community services, focusing on costs, cost-effectiveness and scope of use, compared with EUSCREEN project Country Reports describing how photo- and automated screening is used internationally.

**Methods:**

A systematic narrative review was carried out of all English language photoscreening literature to September 10th 2018, using publicly available search terms. Where costs were considered, a CASP economic evaluation checklist was used to assess data quality.

**Results:**

Of 370 abstracts reviewed, 55 reported large-scale community photoscreening projects. Five addressed cost-effectiveness specifically, without original data. Photoscreening was a stand-alone, single, test event in 71% of projects. In contrast, 25 of 45 EUSCREEN Country Reports showed that if adopted, photoscreening often supplements other tests in established programmes and is rarely used as a stand-alone test. Reported costs varied widely and evidence of cost-effectiveness was sparse in the literature, or in international practice. Only eight (13%) papers compared the diagnostic accuracy or cost-effectiveness of photoscreening and VA testing, and when they did, cost-effectiveness of photoscreening compared unfavourably.

**Discussion:**

Evidence that photoscreening reduces amblyopia or strabismus prevalence or improves overall outcomes is weak, as is evidence of cost-effectiveness, compared to later VA screening. Currently, the most cost-effective option seems to be a later, expert VA screening with the opportunity for a re-test before referral.

## Abstract (zh)

**摘要:**

针对视力降低 (VA) 、屈光危险因素或两者可进行弱视筛查。 VA测试在4岁以下是不精确的, 因此自动风险因素照片的筛选似乎是一个可行的选择。与EUSCREEN项目ʻ国别报告’ (该报告描述了国际上如何使用光筛和自动筛查) 相比, 本评论考虑了社区服务中使用的图像筛查方法, 重点关注成本, 成本效益和使用范围。

**方法:**

截至2018年9月10日, 我们使用搜索词在公众平台对所有英文文献进行了系统性回顾。在考虑成本的情况下, 使用了CASP经济评估清单来评估数据质量。

**结果:**

在370个摘要中, 有55个报告了大型社区图像筛选项目。没有原始数据的情况下, 有五个考虑了成本效益问题。在71% 的项目中, 图像筛选是一个独立的, 唯一的测试项目。相比之下, EUSCREEN 45个国家报告中的25个表明, 图像筛查通常只对既定程序中的其他测试进行补充, 并且很少将其用作独立测试的项目。成本差异很大, 在文献或国际惯例中, 成本效益的证据很少。只有八篇 (13%) 论文比较了图像筛查和VA测试的诊断准确性或成本效益, 而这样做时, 图像筛查的成本效益却不佳。

**讨论:**

与后者VA筛查相比, 图像筛查在降低弱视或斜视患病率或改善总体预后的证据方面 薄弱, 成本效益的证据也很少。目前, 最具成本效益的选择似乎是专家VA筛查, 并有机会在转诊前进行重新测试。

## Introduction

Amblyopia is usually asymptomatic and treatment is much more effective if carried out before the age of about 7 years [1–3], so it fulfils many of the World Health Organisation (WHO) criteria [4] as a target condition for screening. Unscreened prevalence is ~3% [5–7] in Caucasian populations, while that of significant refractive error can be up to 16% [7, 8] because many children with refractive errors will have normal best-corrected visual acuity (VA). Screening of young children significantly reduces amblyopia prevalence [5, 9] so it is recommended or mandated in many countries.

The US Preventative Services Taskforce [10] recommends vision screening at least once in children aged 3–5 years to detect amblyopia or its risk factors, but did not find sufficient evidence to determine the optimal screening interval in these children or to recommend screening under the age of 3 years [11].

It is still unclear how children should be screened to achieve optimal visual outcomes while avoiding excessive costs, false referrals, and unnecessary (or unnecessarily early) treatment. A review by Solebo et al. [12] concluded 4–5 years is the optimum age to offer whole population screening for amblyopia, but explicitly excluded refractive risk-factor screening.

Amblyopia screening either tests for low VA itself, or its *risk factors*, or both. VA testing has proven efficacy [6, 13, 14], but only becomes accurate (e.g. a logMAR linear test with >95% testability) in most children at around 4–5 years of age [15] and requires highly skilled personnel.

If refractive amblyopia risk factors can be detected earlier (especially significant hypermetropia, anisometropia or astigmatism which are the more amblyogenic than myopia), treatment can start quickly and may be easier, and some cases of amblyopia and strabismus, can be prevented altogether. Such better outcomes could aid the development of literacy, motor or social skills.

However, evidence of long-term efficacy of risk-factor screening compared to VA screening in reducing the long-term prevalence of amblyopia is weak. More children will have early risk factors than will ever become amblyopic, because some will emmetropise out of early refractive errors or will accommodate to overcome modest hypermetropia.

Of note, there is still no definitive study that has established the relationship between the size and type of risk factors in early childhood, and the increased odds of developing amblyopia. Clinical consensus based on the best published evidence drives guidelines and definitions of referral thresholds [16, 17] but empirical evidence is scarce.

Screening for refractive risk factors can be semi-automated using photo- or autorefraction and is possible even in infancy. Each test is quicker and less skilled, so cost per screen is low. This, and the potential for earlier referral, might, superficially, be very attractive to commissioners.

Photoscreeners (e.g. Plusoptix™, SPOT™ and GoCheck Kids™) generally test both eyes simultaneously from a distance of 1m. Some detect ocular media opacities and larger angle strabismus but may miss microstrabismus. Autorefractors such as the Retinomax™, test one eye at a time and cannot detect strabismus. There are also smartphone apps that families can use at home. Henceforward, where discussion applies to the principle of automated detection of refractive error in general, the term ʻphotoscreening’ will be used, but when discussing specific studies the precise method will be specified if relevant.

There is a large literature and large-scale marketing advocating phototorefraction as a low-cost option, but costs for photoscreening referrals are generally not loaded at the screening stage. They occur post-referral, often not borne by the screening funders. Overall long-term costs are potentially much higher and difficult to compare with VA screening.

The EUSCREEN Study compares the cost-effectiveness of different vision and hearing screening programmes from 45 countries, mostly in Europe, and presents a publicly available model to assist with implementation, modification or disinvestment of screening programmes worldwide. During the study it became clear that the way photoscreening is being adopted does not reflect, or is necessarily supported by, the literature. Some decisions do not seem to have been based on any evidence of cost effectiveness in comparison to other screening. This report systematically assesses whether the published evidence of photoscreening reflects community adoption in non-research contexts. Where possible we also examined any evidence of costs and cost-effectiveness.

## Methods

The primary aim was to report how photoscreening for amblyopia risk factors is being applied in practice. A further objective was to assess the quality of any reports of costs or cost-effectiveness, whether used as a stand-alone test or when added to other childhood screening batteries.

A systematic narrative review was designed and registered with PROSPERO [18], an international database of prospectively registered systematic reviews in health and social care. Two reviewers (AH, PM) selected the search strategy and terms which are publicly available on the PROSPERO website [18]. Online searches of MEDLINE, EMBASE, PsycINFO via Ovid, CINAHL and The Cochrane Library from 1980 were undertaken on September 10th 2018. The University of Liverpool orthoptic literature database [19] was used to search the British and Irish Orthoptic Journal, American Orthoptic Journal, Australian Orthoptic Journal, European Strabismus Association, International Strabismus Association and the International Orthoptic Congress full paper conference proceedings not electronically listed.

We included full-text original articles in English (i.e. not conference abstracts, opinion pieces, guidelines, editorials). As equipment is usually developed and validated using clinical or community populations, the initial search included all papers reporting photoscreening. After the primary search we included those using, or assessing feasibility of, photoscreening in unselected children under 7 years of age in actual or potential community projects; specifically searching for any papers where costs were explicitly mentioned. AH carried out the primary title stage extraction and excluded publications that were not written in English, letters, editorials, conference or poster abstracts and duplicates. Abstracts from the remaining papers were screened by two reviewers (AH and AC) independently of each other. Non-commercially available methods; test development; validation of tests and new equipment against each other or against other methods of refraction; refractive error prevalence studies; or those deriving or refining referral criteria; and older and non-typically developing populations were excluded. Birefringence scanning for foveal fixation [20], only recently commercially available, was not considered.

Selection was carried out iteratively using the predetermined inclusion/exclusion criteria. Full texts were obtained for the articles considered as relevant, potentially relevant, or if doubt existed. Any disagreements between reviewers were to be resolved by a third researcher at the full paper stage if disagreement remained.

Data extraction for the full-text studies was carried out by AH using a pre-prepared data extraction sheet using criteria agreed by AH and AC. This included, but was not limited to: study characteristics (author, year, country, study type/design); characteristics of the screening programme (including target condition(s), the study sample (e.g. age, gender, ethnicity), tests undertaken as part of the screening, location, and whether on an unselected population or a targeted group. Where reported, outcomes (untestable children, re-tests, sensitivity, specificity, positive predictive value (PPV), negative predictive value (NPV), referral rate, false positives and false negatives) and details of short- and long-term costs were noted. Possible costs were recorded as classified as in Table 1.

**Table 1:** Possible costs of screening to illustrate that cost savings in one funding area may increase unrecognised costs in another.

**Monetary costs**
Equipment; units needed per service, initial purchase, insurance, maintenance and planned ongoing replacement
Admininstration of appointments/information materials/consent process
Consumables
Staff training costs
Staff time to administer test
Monitoring/audit costs
Admininistration of records/databases
Cost of recall/re-test/following-up failed appointments
Referral and outcome feedback costs
Secondary diagnostic referral visit with an eye care professional
Total screening cost per case detected
Cost of follow-up of borderline/untestable children
Glasses
Patches
Total cost per case—referral to discharge
Total cost per false +ve—referral to discharge
Parental time off work
Family travel costs
Lifetime cost-effectiveness (QALYs etc.)
**Non-monetary considerations**
Child/parental anxiety
Bullying/social stigma/psychological
Educational advantage/disadvantage from the condition or its treatment

Cost not just mentioned in passing (statements such a ʻthis is a low-cost method’ in a discussion, but with no supporting data), were evaluated using the CASP Economic Evaluation Quality Checklist [21]. A sample 20% of these were cross-checked by a second researcher (AC). The checklist authors do not recommend a formal score, but suggest it is used as a guide to assess the rigour of the evaluation. Articles were therefore assigned to an ordinal quality scale depending on how many of the 12 items on the checklist had a ʻyes’ response, even if a comment was made such as ʻin part’ or ʻattempted’.

Within the EUSCREEN project, Country Representatives have been collecting national and local data on vision and hearing screening and have completed extensive questionnaires, containing 126 questions on vision screening across 9 domains. Reports were prepared with detailed information on societal background, general screening programmes, vision screening programmes, protocols, outcomes and costs in each country [22]. If photoscreening was being used, the EUSCREEN team requested additional details as necessary.

## Results

The literature search returned 573 potential titles. Two hundred and three were excluded at the title stage. From the remaining 370 papers, 88 were considered within scope, with disagreement on ten further abstracts. These 98 papers were obtained for detailed examination. Both reviewers read the disputed ten papers and after this process one or other of the reviewers changed their opinion independently in the light of additional detail, so a third reviewer was not involved. At this stage 38 were excluded, resulting in 60 papers for inclusion (see Supplementary File for details of exclusions).

Most studies did not report local or national screening schemes as such. In the United States (US), some well-established research groups (e.g. The Alaska Blind Children Discovery (ABCD) group [23–32], groups in Tennessee [33–39] and Pennsylvania [40–48] the multi-centre Vision in Preschoolers (VIP) Group [49–54] have published many papers developing the evidence-base on the most effective photoscreening tests, their validation and refinement of referral criteria. Fifty-five percent of the 370 abstracts reviewed were from the US, 17% from Europe and 14% from East Asia.

### Implementation and outcomes of photoscreening

Fifty-five papers described the implementation and outcomes from large-scale community screening, or screening carried out as a pilot or feasibility studies for potential programmes (see Supplementary Table). Five further papers [20, 55–58] specifically addressed cost-effectiveness from a theoretical standpoint using published data. Thirty-nine papers (71%) reported photoscreening used as a stand-alone test, although the ABCD protocol used an additional brief history and ʻwarning signs’ questionnaire for parents [24].

Only 13 (24%) studies directly compared different test options [29, 49, 55–66]. Four (7%) reported schemes where VA was the primary test, with photoscreening as an adjunct or part of a test battery [65–68]. Of note, Matsuo et al. [65] found adding autorefraction to VA testing at 3.5 years tripled the referral rate, but only increased amblyopia detection by 0.3%, concluding it was not cost-effective. One paper reported photoscreening as a primary test, with further extended screening only who failed [69]. Lowry and Campomanes found that repeating the photoscreening before referral was found to reduce referrals [70].

The age of children tested varied considerably. Many studies tested a wide age range. e.g. 6 months to 12 years [71], 2–9 years [72], 1–5 years [25, 73], 2–6 years [74], 4–7 years [30], 8 months to 5 years [75], 2 and 4.5 years [76], 3–11 years [33] and within these samples, age distribution often also varied. In many developing countries, without established vision screening, charitable outreach photoscreening may only ever be done once per community, so children from infancy to adolescence were screened [77]. Many papers reported ʻpre-school’ children (~3–6 years [24, 29, 50, 64, 65, 78–81], while others specifically targeted infants and toddlers [31, 36, 82–86].

Sixty percent of the papers reported diagnostic ʻsuccess’ in terms of accurate detection of risk factors, not actual amblyopia or low vision, and only 13% reported actual amblyopia detection [24, 57, 59, 60, 64, 65, 87]. The definition of risk factors varied as published guidelines and definitions, such as the AAPOS [16] or the ABCD Group [28] recommendations, developed over time, so comparisons across the years are difficult. Few studies were able to assess the children with significant bilateral hypermetropia (a major amblyopia risk-factor), but who accommodated sufficiently to pass a brief photoscreening [49, 88], but one study found that Plusoptix photoscreening detected only one out of 13 children with significant hypermetropia [60].

The reporting of follow-up rates, and particularly long-term outcomes was often poor or absent. US studies, despite great efforts to encourage follow-up and reporting, often had disappointing follow-up rates of <40% [24, 29, 79, 89, 90]. Good rates of >70% were rare [60, 91, 92], so lack of data is a barrier to any accurate long-term relative risk or cost-effectiveness modelling [58, 71].

Untestable and inconclusive rates in young children were often high. Longmuir et al. [91] reported 25% untestability in 210,695 infants under 12 months and Hope et al. [61] reported up to 24% untestability at 6–9 months. Many screening programmes (especially if administered by lay screeners), would advise a ʻrefer’ or ʻrecall’ decision for untestable children.

PPVs even for risk factors, not actual amblyopia or reduced vision, varied widely from 19% [85] to >80% [27], but were generally lowest in the youngest children [90]. Referral rates were particularly high in very young children e.g. 19% at 6–9 months [84], 20% at 9–36 months [83], 16% at <12 months [61], but often did not result in immediate treatment. One study reported that only 11% of 123 children under 36 months referred received any intervention, compared to a 74% in children over 36 months [93].

### Cost evaluations

Costs were considered in 23 (38%) of the 60 full papers included (Table 2) and were mentioned in passing in a further 16%. Similar findings appear in a paper and extended conference transaction [55, 56] so were considered together. Only four papers addressed more than seven of the 12 CASP checklist items [20, 24, 56, 58]. Even if a checklist item was given a ʻyes’, it was often only very superficially addressed.

**Table 2:** Cost considerations.

Author [references] (ages tested)	Title	Country and setting	Cost defined	Formal cost-effectiveness analysis/modelling	Options compared	CASP checklist items considered	Comments
Arnold et al. [24] (1–5 years)	The cost and yield of photoscreening: Impact of photoscreening on overall paediatric ophthalmic costs	US (Alaska)	Per screen, per RF, cost of adding to current AAP guidelines eye care	Cost-consequence analysis using reference-case analysis over 10 years of life	Cost of adding photoscreening to current recommendations	8	US setting where the AAP/AAPOS guidelines recomment multiple screenings
Arnold et al. [26] (not stated)	Predictive value of inexpensive digital eye and vision photoscreening: ʻPPV of ABCD’	US (Alaska)	Of different equipment	No	Equipment/interpretation cost of different auto/photo methods	1	
Arnold and Donahue [94] (6 months–4 years)	The yield and challenges of charitable state-wide photoscreening	US (Alaska and Tennessee)	Per child screened	No	Comparing cost per screened child in two US states	1	
Cordonnier and Kallay [83] (9–36 months)	Non-cycloplegic screening for refractive errors in children with the hand-held autorefractor Retinomax: final results and comparison with non-cycloplegic photoscreening	Belgium	To diagnosis, including false positives. Per child screened, per child with confirmed risk factors	Estimated cumulative costs to diagnosis	Retinomax vs. photoscreening	5	Enriched population incl higher risk infants, and not community screening.
Donahue et al. [95] (6–47 months)	Lions Clubs International Foundation core four photoscreening: Results from 17 programmes and 400,000 preschool children	US (Multi state) + Taiwan	Per child screened and per child with risk factors detected	No	Programme comparison	3	
Donahue et al. [36] (6–47 months)	Screening for amblyogenic factors using a volunteer lay network and the MTI photoscreener. Initial results from 15,000 preschool children in a state-wide effort	US (Tennessee)	Per screen	No	No	2	
Halegoua and Schwartz [93] (6 months–6 years)	Vision photoscreening of infants and young children in a primary care paediatric office: can it identify asymptomatic treatable amblyopic risk factors?	US (New York)	Per device	No	No	2	
Joish et al. [20] (6 months–8 years)	A cost-benefit analysis of vision screening methods for preschoolers and school-age children	US	Lifetime costs to society	Societal perspective decision-analytic modelling based on published data	VA vs. photoscreening at ages 6–18 months, 3–4 years and 7–8 years	8	Did not consider 5–6 years
Kemper and Clark [118] (3–5 years)	Preschool vision screening in paediatric practices	US	Survey of reimbursement issues as a barrier to screening	No	National reimpursement experiences	n/a	
Kirk et al. [31] (<2 and 2–4 years)	Preverbal photoscreening for amblyogenic factors and outcomes in amblyopia treatment: early objective screening and visual acuities	USA (Alaska)	Estimated extrapolated cost per US child of adding screening to existing services	Partial	Adding screening at 18 months to existing provision. Comparison with comprehensive eye exam costs	5	
König and Barry [56] (3 years)	Cost-effectiveness of screening for amblyopia in 3-year-old kindergarten children: Non-cycloplegic refractive screening with the Nikon Retinomax hand-held autorefractor vs. orthoptic visual acuity screening	Germany	Per case detected	Decision-analytic modelling	Five different screening modalities including photoscreening. Modelled cost of adding tests	6	Only considered screening at 3 years of age in German setting (GPs and Paediatricians)
König and Barry [55] (3 years)	Economic evaluation of different methods of screening for amblyopia in kindergarten	See above	See above	See above	See above	8	Conference transaction paper of above study
Lang et al. [32] (3–11 years)	Validated portable paediatric vision screening in the Alaska Bush. A VIPS-like study in the Koyukon	US (Alaska)	Per screen	No	No	1	Small local study
Leman et al. [62] (3–7 years)	A comparison of patched HOTV visual acuity and photoscreening	US (Alaska)	Per test	No	No	3	
Longmuir et al. [92] (6 months–6 years)	Nine-year results of a volunteer lay network photoscreening programme of 147,809 children using a photoscreener in Iowa	US (Iowa)	Per child screened	No	No	2	
Lowry et al. [68] (31 months–6 years)	Efficient Referral Thresholds in Autorefraction-Based Preschool Screening	US (California)	Cost per case detected screening and follow-up visits, but not glasses prescriptions	Retrospective evaluation. Modelling to arrive at optimum referral criteria towith minimal cost	Different referral criteria for same photoscreener	6	Optimal model verified with follow-up study. Cost from persective of ʻ3rd party care providers’
Lowry and Campomanes [57] (31 months–6 years)	Cost-effectiveness of School-Based Eye Examinations in Preschoolers Referred for Follow-up From Visual Screening	US (California)	Cost per case detected at follow-up funded a service	Decision-analytic modelling and probabilistic sensitivity analysis	Community vs. mobile follow-up	3	Modelling costs of different types of follow-up, not screening itself
Lowry et al. [80] (3–4 years)	Repeat Retinomax screening changes positive predictive value	US (California)	Cost savings made by re-screening	No	Single screening vs. repeat before referral	4	
Matsuo et al. [65] (3.5 years)	Is refraction with a hand-held autorefractometer useful in addition to visual acuity testing and questionnaires in preschool vision screening at 3.5 years in Japan?	Japan	No	No	Value of adding photoscreening to established programme	1	Not cost effective
Miller et al. [63] (3–5 years)	Cost-efficient vision screening for astigmatism in native american preschool children	US (Arizona—native American)	Cost of running service/case detected (but not technician time or consumables)	Modelled cost in different size populations to be screened	Four different screening modalities	4	Population with high astigmatism risk. Refrral criterion was ʻenough astigmatism to warrant glasses’
Morgan and Kennemer [96] (5, 7, 11 years)	Off-axis photorefractive eye screening in children	US (East Coast states)	Cost per screen and cost per affected child (incl ref. error)	No	No	2	Early paper
Rein et al. [58] (3 years)	The potential cost-effectiveness of amblyopia screening programmes	USA	Programme cost, referral rate follow-up data	Yes. Randomised person-level simulation. Probablistic sensitivity analysis	Three different screening scenarios including photoscreening at 3 years + VA screening at 5 years	9	All screening modalities likely to be cost effective compared to other public health programmes. The scenario involving photoscreening was most costly. Did not assess photoscreening as a stand-alone test
Terveen et al. [71] (6 months–12 years)	Results of a paediatric vision screening programme in western South Dakota	USA (South Dakota)	Cost for a State run service. Cost of lost earning power of of undetected amblyopia. Cost ratio of cost to QALY	Cost-utility reference-case analysis	SPOT screener compared to no screening	3	

The CASP checklist score only notes the number of checklist items that were addressed in the paper, not the quality of the evidence.

If actual costs were reported, they varied widely (see Table 2). Nineteen papers reported from an actual screening programme, while the remainder used modelling from data from other sources. Only three of the 23 papers were from outside the US (Germany [56], Belgium [83] and Japan [67]) and six papers presented data from non-typical settings e.g. Alaska, with a remote, sparse population [24, 26, 31, 32, 62] or from a minority ethnic population at higher risk of astigmatism [63].

Most only considered immediate costs: per test [24, 26, 32, 36, 94–96], per case detected (usually cases of risk-factor, not amblyopia) [25, 56, 57, 63, 70, 83, 95, 96], or to diagnosis [56, 57, 63, 70, 83, 95, 96]. Longer-term costs were more disparate: savings made by re-screening [57], screening service cost [58, 63, 71], total cost to a state [73], additional costs of adding photoscreening to established services [24, 31, 56, 65], costs of different types of follow-up [57], costs to third-party providers [24, 57, 63, 70], cost for detection plus follow-up [57, 58, 70], lifetime costs e.g. Quality Adjusted Life Years (QALYs) [20, 31, 71]. Cost-effectiveness was the primary focus of only seven papers [20, 24, 55–58, 63]. None addressed any potential cost benefits associated with the earlier detection of wider refractive error (as opposed to amblyopia) and any possible societal or educational benefits from its correction. Regular maintenance and replacement of relatively expensive photoscreeners within long-term services were not considered.

Eight papers attempted formal modelling [20, 24, 56–58, 63, 70, 97]. Joish et al. [20] considered lifetime societal costs in the US, using societal perspective decision-analytic modelling based on published data, comparing VA screening versus photoscreening at three different ages: 6–18 months, 3–4 years and 7–8 years. All scenarios were considered cost-effective, with total net cost benefit at 3–4 years being highest from photoscreening, but the cost-to-benefit ratio was highest for VA screening at the same age. An important limitation was that testing at 5–6 years was not considered, although this is when VA testing is accurate, but children are still within the critical period. They discussed the methodological difficulties in using modelling approaches when evidence of the societal significance of unilateral amblyopia or ʻpoor’ versus ʻgood’ eyesight (which presumably includes uncorrected refractive error) is weak.

König and Barry [55, 56] used German data and healthcare settings in a decision-analytic model (Monte Carlo simulation) comparing five different amblyopia screening modalities at age 3 years (different VA testing ± orthoptic tests, and non-cycloplegic autorefraction) from a third-party payer perspective, although they did not model beyond diagnosis, or at different ages. They concluded that ʻ*because of a great proportion of false negative, false positive and inconclusive results, refractive screening was less effective with an unfavourable cost-effectiveness*’. This paper is important because most literature agrees that VA testing at 3 years of age can be imprecise, but even so, autorefraction led to much higher cost per case of risk factors detected (≈1500 Euros (€) versus €≈900 for VA screening). They found that adding orthoptic tests to VA testing made only small improvements in detected case numbers. For all scenarios, re-screening inconclusive results was more cost-effective than direct referral after a first screening.

Rein et al. [58] used two US state screening programmes in probabilistic microsimulation modelling considering lifetime per-person costs and QALYs gained. They compared VA/stereopsis (VA/SV)) (Random Dot E 540″ pass/fail) screening at kindergarten (4–6 years) only; similar screening twice at preschool and kindergarten; and photoscreening in preschool and VA/SV screening in kindergarten, but did not consider photorefraction as a stand-alone test. All scenarios were considered cost-effective compared to no screening, with increased QALYs. Earlier photoscreening followed by later VA/SV screening was the costliest option, for both amblyopia-related treatment, and lifetime costs, but it might result in greater long-term benefits than VA/SV screening in preschool alone. Because evidence of actual QALYs lost from amblyopia [98] is sparse, they modelled various scenarios and found wide variations in QALYs driven by different QALY weights and differences in treatment efficacy. Replacing VA/SV in kindergarten with photoscreening in the two-screenings scenario was the costliest option, and only became more favourable when the assumption of QALYs lost per year of impairment was increased.

Miller et al. [63] used an economic model to estimate different screening modalities, (including photoscreening), among preschool children, when different size groups were screened. They only considered the direct costs of the screening and equipment itself, not of longer-term diagnosis, treatment or follow-up. VA screening was more cost-effective for small populations, but automated methods became relatively cheaper for larger groups. Although their target group was a native American population with a high prevalence of astigmatism, their conclusion that photoscreening alone was generally not cost-efficient is likely to also apply elsewhere.

Lowry and Campomanes [70] modelled the most cost-effective autorefraction referral criteria to use in ʻpre-schoolers’ (presumably 3–5 years) across different refractive error risk-factor types. They compared the VIP criteria [88], a local variation based upon them and referral criteria derived from their modelling which would pass more mild refractive errors. They concluded that their own criteria (e.g. up to 3.25 D of anisometropia compared with the VIP criterion of 2.00 D) would be more cost-effective, but they stopped short of considering treatment costs. They also pointed out that many factors beyond their analysis might affect policy decisions in different communities and healthcare models. They subsequently used a retrospective cohort cost-effectiveness and a decision-analytic model and probabilistic sensitivity analysis of referrals from both VA and photoscreening in Californian pre-schools [57]. They concluded that community-based follow-up of referrals was a more cost-effective than mobile clinics but did not analyse the costs of the screening itself.

Arnold et al. [24] reported data from the Alaskan ABCD programme using the MTI photoscreener. They used a deterministic model for their simulation and considered the societal cost per child with amblyopia risk factors over the first 10 years of life. They used a cost-consequence method using Reference-Case Analysis for photoscreening against two established screening paradigms; the 1995 American Academy of Pediatrics guidelines [99] which recommended multiple assessments versus a ʻtrickle in’, unscreened, situation, as well as estimates of costs of other US care options. Cost per amblyopic child was ~40% higher in rural communities, but overall, adding photoscreening increased the (then) current screening costs by 9%, while complete pre-kindergarten eye tests from an ophthalmologist would add 49% to overall eye care US-wide. While clearly supporting the use of photoscreening the authors did acknowledge that there are still many unknowns, particularly in relation to the societal significance of unilateral amblyopia.

Membreno et al. [97] used a cost-utility analysis from a third-party insurer perspective using a decision analysis modelling strategy to estimate costs of undetected amblyopia to a US state as evidence to support a new photoscreener programme. Amblyopia screening was considered cost-effective in terms of QALYs compared to no screening, but they did not compare alternative screening options.

### EUSCREEN country report data

Twenty-five of the 45 countries (56%) returning the questionnaires [100] stated that photorefraction or automated refraction was used to some extent in their country, but national or large regional schemes utilising universal photoscreening were unusual (e.g. Flanders in Belgium). Country Representatives often struggled to access data which was rarely publicly available. Fifteen countries used Plusoptix photoscreening devices and the others used different (sometimes undefined) autorefractors such as Retinomax, or a combination of methods.

Only Israel and Hungary reported that photorefraction or automated screening was widely used as a stand-alone test and in most countries it is administered by experienced professional screeners. Most countries reported that beyond neonatal screening, at least one other test is carried out, generally involving a VA test and/or cover testing and external examination. Photoscreening is often a recent addition to existing services and there was no evidence that since the advent of photoscreening, existing services had been reduced or modified. Audit data was rarely available or collected centrally. The Flanders region of Belgium seems an exception [101] and wide within-country variation was the rule. No country guidelines have mandated photoscreening alone.

Photoscreening is often used to target young children before VA tests are accurate; or for children untestable or equivocal with a VA test; or only for private patients; or in one case, only on older children looking for developing myopia. Ages varied widely: 6 months (1 country), 12 months (2 countries), 18 months (1 country), 3 years (5 countries), 4 years (2 countries), and unspecified times (13 countries). In some countries it was used repeatedly in multiple screenings.

The weight given to the photoscreening also varied: one country reported that if VA was within normal limits, but photoscreening suggested a refractive risk-factor, the child would still be referred. Elsewhere a child passing a VA test would not be photoscreened at all, so would never be referred for risk factors alone.

Photoscreening was used more widely if screening was carried out by non-eye-specialist screeners, particularly paediatricians or GPs e.g. Germany and the Czech Republic. Where private providers carried out screening, photoscreening was more common and could incur additional charges. Where it is offered by doctors with a higher level of expertise and autonomy, the country representatives found it more difficult to access data, and how photorefraction is used is more subject to professional discretion.

## Discussion

Photoscreening is being widely adopted, and in many different ways, but with poor availability of local, regional or national protocols, audit or monitoring of long-term outcomes or costs. There is weak evidence of optimum timing, frequency, or referral criteria to maximise outcomes whilst minimising monetary and societal costs.

Despite published guidelines [16] there is still no clear evidence what level of refractive error constitutes an amblyopia risk-factor at different ages, or the optimum time to treat risk factors. Commissioners have little evidence on which to base difficult public health decisions and may be unaware of the significant differences, in terms of relative outcomes, costs or cost-effectiveness, between VA screening and photoscreening. Different reporting metrics make comparisons very difficult. Issues arising from this review highlight the relative advantages and disadvantages of photoscreening compared to more conventional VA testing, and some have significant cost implications.

The literature gives an impression that photoscreening is commonly used as a stand-alone test, but the EUSCREEN data shows that it is usually an addition to established screening tests.

### Early vs. late screening

The true prevalence and severity of amblyopia in very young children is unknown because a definitive diagnosis is not possible until VA can be tested, so risk-factor versus amblyopia prevalence cannot be compared. The low treatment rate in children under 36 months found by Halegoua and Schwartz [93], suggests poor correlation between risk-factor and amblyopia diagnoses.

Photorefraction is more testable than VA under 4 years of age, so providing an opportunity for early intervention, and is often thus promoted and marketed. The EUSCREEN study shows it has been added to several existing screening programmes, often for the younger age groups, but in different ways and rarely adopted nationally.

Early detection, treatment and possible prevention [7, 102] are generally considered a good thing. However, there are also higher false positive, equivocal referral and untestable rates in younger children [82, 83, 103] and untestable children are often automatically referred [60]. Test–re-test variability can be high and differ between risk factors [80, 104], but decisions are often based on single tests.

Early referral, however, carries considerable additional costs to health services, insurers and parents: more specialist visits and glasses between referral and discharge, parental work absence and travel costs. More children will be referred without a genuine problem, and who may not develop one. PPVs are lower for screening in infancy [103]—even for risk factors, let alone amblyopia. Early referral may not mean earlier (or any) treatment if those on treatment are only observed before a decision to treat or discharge is made [93]. Astigmatism in very young children is a particular problem because it can resolve or change significantly in the first years of life [105].

Evidence from Flanders in Belgium and Portugal shows that early photoscreening can result in many more young children being given glasses [82, 101]. In Flanders, the number of 4-year olds wearing glasses rose from 4.7% in 2012 to 6.4% in 2017 after the introduction of photoscreening but it is unknown how many cases of amblyopia were prevented. Halegoua and Schwartz [93] found that only 3.5% of the 8.5% failing photoscreening with amblyopia risk factors had more than ʻmild’ amblyopia.

Most ophthalmologists would feel professionally bound to follow referred children carefully, and prescription of glasses might take place at a lower threshold than the risk-factor referral criteria. Risk-factor thresholds and prescribing norms are arrived at from average values from surveys of individual prescribing practices [106–109], and can differ widely between professionals in the same country [107]. Diagnostic and prescribing decisions are rarely centrally regulated [103] or recorded. For example, a 3-year-old child may be referred with a photoscreened anisometropia of 2.25 D, which on cycloplegic refraction is found to be 1.75 D. Many professionals would give this child glasses, or at least observe them carefully once referred, even if the anisometropia might not now quite reach the risk-factor referral threshold of 2.0 D difference. Other infants may emmetropise, but still be watched for years and only discharged once VA is testable. Parental anxiety, loss of trust, and costs of repeated follow-up for children eventually discharged without treatment carry societal costs. Professionals in stretched and scarce services do not want multiple false or borderline referrals.

Accurate VA screening requires highly skilled testers, longer testing times and is still imprecise under 4 years of age. Referral may be later and outcomes may be worse in terms of final VA [13], greater overall amblyopia prevalence, longer occlusion times [3], more decompensated strabismus [110], but Donohue et al. [95] reporting experiences of photoscreening from over 400,000 photoscreenings, argue ʻ*Since a small delay in detecting amblyopia probably has minimal to no effect on treatment, a legitimate argument can be made to demand extremely high specificity (i.e. 97% or more) for all vision screening instruments used in healthy preschool populations*’. Such high specificity (and for risk factors, not amblyopia itself), suggested by Donohue et al. is rare [32, 77]; most studies report much lower [59, 61, 79, 85, 89, 92]. Although autorefraction aged 2 can be the best predictor of mild-moderate visual impairment aged 4.5 years, the PPV is generally poor and it does not reduce the prevalence of problems detected later [76].

The *significance* of slightly poorer outcomes, as long as treatment still starts within the critical period (under about 7 years of age), is weak. Kirk et al. [31] describe one logMAR line better from earlier detection (<2 years vs. 2–4 years) as ʻsubstantial’, but the adverse effects of modest delay in starting occlusion under the age of 6 years are less dramatic than beyond 7 years [3]. Following a nationally advised [111, 112] change from health centre tests at 3–4 years, to school entry testing at 4–5 years, national audits by the British & Irish Orthoptic Society [113, 114] suggest that it did not result in worse overall visual outcomes but increased the coverage and accuracy of the screening dramatically.

Evidence that small visual deficits (especially mild unilateral amblyopia) carry increased lifetime costs is weak, although falling below certain thresholds e.g. for driving standards, or extra educational support, might have significance for a few. The evidence that one or two years delay in starting treatment, provided it is before 7 years of age, is surprisingly sparse and sometimes equivocal [31, 115]. Carlton et al. [98] found poor evidence of long-term impact of unilateral amblyopia and little research has been carried out on the relative impact to families of early vs. late referral if outcomes are comparable.

### Financial considerations

Photoscreening may be easy, quick and cheap, but longer follow-up [116] and glasses are expensive [117]. Accurate VA screening takes longer and needs expert testers who cost more, but savings are likely post-referral because PPV is higher and treatment time reduced. Later referral generally means at least 1 year’s less hospital or ophthalmologist follow-up. Although it may mean patches worn in school, many families find this easier anyway.

Cordonnier and Kallay [83] estimated that false referrals of children between 9 and 36 months, and only up to the first diagnostic visit, inflated the cost per screened child significantly. Donohue and Johnson [103] question the justification for referral for astigmatism in children under 2 years of age due to high untestable and referral rates and low PPV. These are significant considerations in countries where access to specialist services is limited by distance, ability to pay, or workforce availability. Kemper and Clark [118] reported that most US family physicians would be unlikely to adopt photoscreening if false positives were over 10%. A 2016 review does not recommended photoscreening [119].

Hybrid and multiple screening services, using photoscreening for the youngest or selected children, followed by acuity testing when older, may load costs onto both screening and treatment stages so adding photoscreening to other testing can be the least cost effective option [56, 58, 65]. Hybrid schemes would only be justified if they led to better outcomes. Any state-funded support for the whole patient journey enforces hard choices, as with the UK National Health Service [12] which has opted for a single VA screen at 4–5 years. In the private medicine context, insurers increase premiums if total treatment costs increase.

### Technical aspects of photoscreening

Papers rarely report other disadvantages of photorefraction. It may miss nystagmus, optic nerve or retinal pathology, or small angle strabismic amblyopia, which VA testing would detect. It is known to be least accurate in detecting and quantifying hypermetropia [60, 120, 121], a major strabismus and amblyopia risk factor [122]. Anisometropia can be missed by uniocular autorefraction if children accommodate, and myopia can be overestimated [79, 123]. Eyelashes or the lids obscuring pupil margins can return false astigmatism or anisometropia readings, and children with particular combinations of iris and retina pigmentation may be untestable. There are also differences in linear operating range with large pupils [122] and in calibration factors between ethnic groups [124]. Commercially sensitive software algorithms derived from largely Caucasian populations may not apply globally and although referral criteria can often be adjusted, background calculation of refractive error cannot.

### Target condition

A final issue is a lack of consensus about what the target condition for vision screening should be [50]. Traditional screening literature has targeted amblyopia and low vision, but much of the photoscreening literature reports ʻsuccess’ as detection rates for *risk factors* for these conditions, not the conditions themselves, with few comparisons [68].

There has been ʻmission creep’ from screening for amblyopia and low vision into screening for significant refractive error, whether amblyogenic or not. Refractive error may be a public health issue itself, but most children with milder refractive errors will not be amblyopic, while some amblyopes will not have significant refractive error.

Detection of early refractive error seems desirable, especially in countries where myopia or astigmatism are major public health concerns [125], especially now that myopia treatments are available [126, 127]. But does refractive error fit WHO criteria for screening? It is largely unpreventable and for many children it may not cause significant harm if corrected a little later. There is some evidence that treatment of hypermetropia might improve visual and educational outcomes [13, 128], but photoscreening is *least* effective for detecting hypermetropia [60]. Can all health services cope with high numbers of mostly mild visual deficits?

### Summary

There is no doubt that amblyopia should be detected before 7 years of age, beyond which it becomes much harder to treat, but from a public health viewpoint, the optimum timing and nature of that screening is still not established. Photoscreening complicates the issue by changing the target condition from amblyopia to amblyopia risk factors, and by allowing earlier testing. Currently, the most cost-effective option seems to be a later, expert VA screening with the opportunity for a re-test before referral [56].

Countries must decide what fits their population, healthcare goals, workforce and funding models. There is a need for clear evidence that the extra post-screening costs incurred by photoscreening for risk factors, especially if carried out on infants and very young children, are justified by improved overall outcomes. Despite efforts to standardise reporting [17], this is still not happening and it is a major barrier for decision-makers trying to making sense of the evidence. The EUSCREEN project aims to support these decisions in the future.

## Supplementary information

Supplemental file 1 Flow chart of review

Supplemental File 2
