# Supplementary figures and images for: Scope and costs of autorefraction and photoscreening for childhood amblyopia—a systematic narrative review in relation to the EUSCREEN project data
Source: Eye (Lond). 2020 Nov 30;35(3):739–52. doi: 10.1038/s41433-020-01261-8 (PMC8026636; doi:10.1038/s41433-020-01261-8)

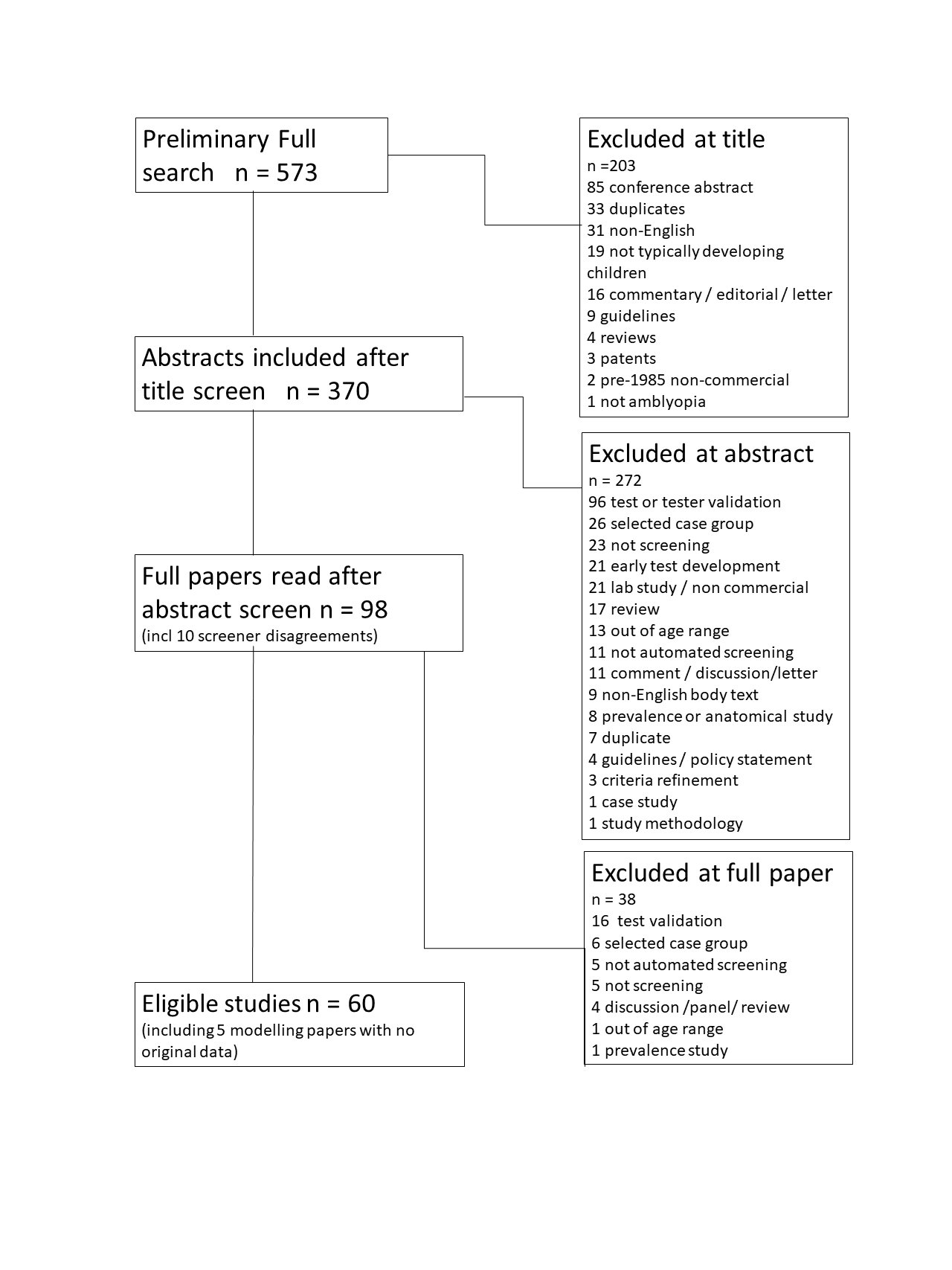

Supplement: Supplementary file 1 — Supplemental file 1 Flow chart of review [file 41433_2020_1261_MOESM1_ESM.tif]
