# Supplementary material for: Scope and costs of autorefraction and photoscreening for childhood amblyopia—a systematic narrative review in relation to the EUSCREEN project data
Source: Eye (Lond). 2020 Nov 30;35(3):739–52. doi: 10.1038/s41433-020-01261-8 (PMC8026636; doi:10.1038/s41433-020-01261-8)
Supplement: Supplementary file 2 — Supplemental File 2 [file 41433_2020_1261_MOESM2_ESM.pdf]

**Data extraction sheet for the 55 papers included in the review**	Purpose	Country	n	Target condition	Age tested	PhotoS used as primary screening test	Which photscreener	PhotoS used alone or in battery	How many followed up after screening	Referral rate	Intestable /inclon-clusive	Cost mentioned at all	PPV if available
Arana Mendez, M, et al (2015)	Primary photoscreencompared with with full exam in local context	Costa Rica	219	Risk factors	2-9 yrs	Yes	SPOT	Alone	all followed as part of study	19.60%	1.3% (all aged 2/3)	no	58.10%
Arnold, RW (2003)	Feasibility study using state fair screening data	US (Alaska)	4343	Risk factors	3.5yrs (+/- 1.5yrs)	Yes	MTI	Alone	2.80%	7%	n/a	In passing	>90%
Arnold, RW, et al (2005)	Cost effectiveness estimation using data from 7 years of community screening	US (Alaska)	13255	Risk factors	1-5yrs	Yes	MTI	Alone	38.00%	13%	0.50%	Yes	>90%
Arnold, RW, et al (2007)	Proof of concept and validation study of MTI with other digital cameras	US(Alaska)	2900	Risk factors	"school children"	Yes	MTI + other digital cameras	Alone	39%	6-11%	0.50%	In passing	84%
Arnold, RW, and Donahue, SP(2006)	Comparison of two state-wide schemes	US (Alaska & Tennessee)	14,000 and 100,800	Risk factors	mainly pre-school (3-5yrs)	Yes	MTI	Alone	Tenn 72% Alas 49%	n/a	n/a	Yes	73-89%
Arnold, RW, et al (2000)	Early report of photoscreening experience	US(Alaska)	3930	Risk factors	0-14yrs (mainly "preschoolers")	Yes	MTI	Alone	43% confirmed +8% by report	8.90%	No	In passing	77-92%
Arnold, RW, et al (2008)	Compared VA tesing with photoscreening, incl. time to test	US (Alaska)	1700	Risk factors really present	4-7yrs	VA and photoscreening compared	MTI, Gateway DV-S20	Both, as part of comparative study	14%ful exams traced	13% (incl "incomplete")	Approx 0.7%	No	74-100%
Arnold, RW, et al (2012)	Comparing new/old photoscreeners in paediatric practices	US (Alaska)	675	Risk factors	1-4yrs	Yes	Plusoptix SO9	Alone	48%	12%	n/a	no	69% (but only 9% for strabismus)
Arthur, BW, et al (2009)	Pilot / feasibility comparing photoscreening with later full exam	Canada	307	Risk factors	4-5 yrs	Yes	Plusoptix SO4	Alone (full exam later as validation)	n/a	9.40%	1%	No	73%
Asare, A O, et al (2017)	Pilot /feasibility as funding argument	Canada	1321	Risk factors	1-5yrs	Yes	Plusoptix S12C		50%	6.10%	3%	No	81.80%
Barry, JC and König, H H (2001)	Feasibility study. Comparing photsceeening with orthoptic sreening.	Germany	404	Amblyopia	3yrs	n/a	Retinomax	Both as comparison	n/a	43% including untestables inclonclusive	26%	No	73%
Carneiro, I, et al (2018)	Pilot for national scheme	Portugal	2867	Stated "amblyopia" but reported risk factors	2 yrs	Yes	Plusoptix SO9	Alone	55%	18.00%	no	No	58.40%
Chang, DA, et al (2015)	Local feasibility study of photoscreening	US (Hawaii, high ethnic minority population)	137	Risk factors	8mth- 5yrs	Yes	Plusoptix S12	Alone	40%? and incomplete	8% refer	13%	No	n/a
Chen, X, et al (2016)	Prevalence ambly & strab in 3-6 yr olds.	China	5884	Amblyopia	36-72 mths	No	Not stated	Full test battery	Not stated	Not stated	Not stated	No	n/a
Clarke, N, et al (2008)	School nurse pilot project in a general health screening battery	US (California,86% hispanic)	592	Risk factors	2-5yrs	Yes	Suresight	Alone	69% traced (only 17% intended to seek treatment)	7%	1.30%	No	n/a
Cordonnier, M and Kallay, O.(2001)	Test validation study of an higher risk sample. Retinomax vs older photoscreeners + cost modelling	Belgium	1218	Risk factors	9-36 months	n/a	Retinomax	n/a	n/a	19.60%	n/a	yes	19% anisometropia 55% hypermetropia 69% astigmatism
Couser, NL (2014)	Report of community Plusoptix screening	US	15075	Signif ref error incl. risk factors	4 yrs	Yes	Plusoptix SO9 and S12	Alone	30% reported seeking advice. No validated data	24-28%	n/a	in passing	n/a

Dahlmann-Noor, AH, et al (2009)	Comparing orthoptic screening with photoscreening	UK	288	Amblyopia	4.5-7.3 yrs	Masked comparison with orthoptic screening	Plusoptix CR03	Masked comparison with orthoptic screening	85%	5.60%	2.40%	No	n/a
Darusman, KR(2014)	Pilot community project	Inodonesia	166	Risk factors	2-6yrs	Yes	Plusoptix AO9	Initially alone	n/a	15.67%	<1%	no	n/a
Donahue, SP, et al (2006)	Large composite dataset from 15 Lions clubs projects	US mulit-state + Taiwan	400,000	Risk factors	3-5 yrs	Yes	MTI	Alone	mean 54% (range 7-70%)	mean 5.2% (range 3.7-12.6%)	3-14%	yes	80%
Donahue, SP and Johnson, TM (2001)	Refinement of referral criteria		31,053	Risk factors	<4yrs	Yes	MTI	Alone	57-73%	7.8% under 1yr, 3.8% >5yrs	12% under 1yr, 1-2% at 4-5 yrs	in passing	38% under 1yr, 73-76% at 4-5 yrs
Donahue, SP and Johnson, TM (2000)	Report of statewide project	US (Tennessee)	15,000	Risk factors	6-59mths	Yes	MTI	Alone	57% but 7% of these already being treated	9.8% under 1yr, 7% at 5 yrs	10.2% under 1yr, 3.3% at 5 yrs	yes	60.30%
Dostalek, M and Benesova, J (2002)	Recruitment issues around infants tested by paediatric GPs	Czech Rep	780	Risk factors	6-9mths	Yes	Early bespoke photoscreener	Alone	n/a	19.00%	n/a	no	n/a
Goodman, L., et al (2018)	Longitudinal comparison of screening at age 2 (incl.autorefraction), with VA tests at 4.5 yrs	New Zealand	355	Reduced VA at 4.5yrs	2 and 4.5yrs	Yes, but part of battery at 2 yrs	Suresight	Battery at 2 and 4.5yrs	n/a	10.1% at 2	73% for autorefraction	no	46%
Halegoua, J and Schwartz, RH (2015)	Feasibility & accuracy of multiple early screenings	US, New York	1976	Risk factors	6mth-6 yrs at annual checks	Yes	SPOT	Alone	56% sought care, only 3.5% amblyopia beyond "mild"	8-16%	n/a	yes	27% (excluded known previous diagnosis)
Hendler, K, et al (2016)	Report of charitable photoscreening servce outcomes	US (California)	11260	Risk factors & ref error	3-5 yrs	Yes	Retinomax	Alone	65%	16%	n/a	in passing	74% "for a need for glasses" not amblyopia or risk factor thresholds
Hope, C, et al (1994)	Early feasibility study on new device	New Zealand	278	Risk factors in infants	6-9 mths	n/a	Aukland	Alone for this context	75%	16% at <1yr	19-24%	in passing	<27%
Huang, D, et al (2017)	Mainly test validation, but incl. screening data	China	1818	Risk factors	3-4yrs	Yes	Plusoptix A12C	Alone	20.24%	3.90%	3%	no	40% (AAPOS criteria)
Kemper, AR. and Clark, SJ (2006)	Scope of screening offered by US paediatricians.Comparable to Euscreen survey	US (Michigan)	n/a	n/a	n/a	n/a	n/a	n/a	n/a	n/a	n/a	Yes	n/a
Kemper, AR, et al (2005)	Comparing monocular autorefractor with full eye exam,	US (Michigan)	170	Ref. error, strabismus, amblyopia	0-5yrs	n/a	Suresight	Alone	36%		51% under 3 yrs	no	n/a
Kirk, VG, et al (2008)	Follow up study comparing outcomes of children detected before /after 2yrs	US (Alaska)	21,367	Risk factors	under 2 vs. 2-4 yrs	Yes	MTI	Alone	6.3% followed in detail	6.90%	n/a	in passing in disc	82%
Kulp, MT (2009)	Definitive report fro VIP group comparing many screening modalities.	US (multi state)	2588	3 levels of severity of impairment	3,4,5 yrs	Comparitive analysis of many tests /personnel	Retinomax, MTI, Suresight, Plusoptix	All combinations analysed	all	n/a	2%	no	n/a
Lai, YH, et al (2013)	Study of the best combination of VA, autor ref & SV (main focus is myopia detection)	Taiwan	1000	Risk factors	2.5-7.3yrs	Battery,VA, SV, photos	Topcon autorefractor	Battery	all	7%	n/a	no	33%
Lang, D, et al (2007)	Small local project trying to emulate VIP study in rural area	US (native Alaskan)	80	Risk factors	3-11yrs	Yes	Suresight, MTI, Gateway	Alone	none	12-15%	21%	yes, but only cost per test	63-94%
Leman, R, et al (2006)	Comparing VA testing for amblyopia and photscreening for risk factors	US (Alaska)	1667	Risk factors but NOT low VA due to ref error	"pre kindergarten to 1st grade"	Comparative study with "enhanced" VA testing	MTI & Gateway	Comparative study	14%	n/a	no data but discussed	yes	83-91%
Longmuir, SQ, et al (2013)	Large cohort study of pre-school photoscreening	US (Iowa)	210695	Risk factors	6-48 mths	Yes	MTI	Alone	76%	3.30%	25% under 1yr, 13% in under 3's,4.1% 3-5's	no	86.8% for risk factors, similar across all ages
Longmuir, SQ,et al (2010)	Large statewide project results	US (Iowa)	147809	Risk factors	6/12 to 6yrs	Yes	MTI	Alone	>80%	4.4% referred + 5% unreadable	unreadable 10-20% under 2	yes	>90% for all risk factors

Lowry, EA and Campomanes, A (2015)	Formal modelling of most efficient ref criteria from perspective of healthcare provider	US (California)	6333	Risk factors	31mths-school age?	Yes	Retinomax	Alone	65%	10.70%	n/a	yes	n/a
Lowry, EA and Campomanes, A (2016)	Comparison of cost per detected case between 2 different type of follow up	US (California)	1524	Amblyopia	3-5yrs	Yes but also compared with an earleirVA tested group	Retinomax	With CT and light rreflex	59%	5.10%	no	yes	n/a
Lowry, EA, et al (2014)	Comparing referral rates with and without a recall	US (California)	7614	Risk factors	pre-school (mean 4.3yrs)	Yes	Retinomax	Alone + retest	48.80%	17% (27% of 2nd screen so 9.6% finally referred)	12% with plusoptix	yes	n/a
Lowry, EA, et al (2015).	Comparing referral rate changes in 3 yr olds before and after adding Plusoptix. Testability at age 3	US (California)	1551	Risk factors	3	No (VA 1st then Plusoptix if examiner chose)	Plusoptix	Battery	47%	increase of 80% after introduction of photoS	12%	no	51% for risk factors, 41% for potential amblyopia
Matsuo,T,et al (2009)	Looked at cost of adding photoscreening to existing service	Japan	265	Amblyopia & pathology	3.5	No	Nidek	Battery	58%	x3 what would have been if just VA tested for 0.3% increase in amblyopia detection	6%	yes	n/a
Matta, NS, et al (2010)	Comparing Plusoptix with full eye exam. Charitable outreach.	Honduras	105	Risk factors	up to 17 yrs (few young children)	Yes	Plusoptix SO4	Alone vs full exam	all	13%	n/a	no	n/a
Mehravaran, S, et al (2016)	Primary photoscreening with full on-site exam for photoscreen fails	US (California)	12,088	Ref error and amblyopia	3-5 yrs	Yes	Retinomax	Alone first	64%	16% only 0.8% amblyopic, most others just ref error	no	no	n/a
Miller, JM, et al (2003)	Detection of astigmatism in a higher risk group + cost effectiveness	US (Arizona native American)	167	Astimgatism	3-5 yrs	Test comparison VA, MTI, Retinomax, keratometry	n/a	Battery	n/a	n.a	no	yes	n/a
Moghaddam, AA, et al (2012)	Pre verbal screening in community setting	Iran	996	Risk factors	6-36 mths	Yes	Plusoptix	Alone	67%	47.80%	1.90%	in passing	19.2% for amblyopia
Morgan, KS and Kennemer, JC (1997)	Early study, non-commercial equipment	Canada	14075	Risk factors and strabismus/pathology.	5,7,11yrs	yes	Bespoke camera	Alone	n/a	11.30%	3.50%	yes	n/a
Panda, L, et al (2018)	Multi stage screening . VA by teachers then later refraction	India	153,000	Reduced vision	5-15 yrs	tested independently from VA	SPOT	Cmparative study	only fails	5.4% (incl. many older children)	n/a	in passing in conc	n/a
Peterseim, M M, et al (2015)	Plusoptix community screening + teacher survey of treatment acceptance	US (South Carolina)	2750	Risk factors	3-5 yrs	yes	Plusoptix SO8	Alone	56%	27%	no	no	46%
Ransbarger, KM, et al (2013)	Report of community screening	US (California)	7814	Risk factors	6-72 mths	yes	SPOT	Alone	12.50%	31%		no	65.70%
Ruao, M,et al (2016)	Photoscreening in 1yr olds	Portugal	11245	Risk factors	12-18 mths	yes	MTI & Plusoptix	Alone	77%	6.5% + borderline 12%	2.00%	no	56.80%
Savage, HI, et al (2005)	Testability/ reliability & learning curve study	US (Washington DC)	200	Amblyopia	3-6 yrs	no	Suresight	battery VA+ SV+autoref	68%	n/a	2-11% worse if younger	no	n/a
Schaeffel, F, et al (2007)	Part of large scale longitudinal validation study	Germany	736	Ref error	1-2 yrs	yes	Power Ref 1 +/- +3.00 lenses	Alone	n/a	11.50%	n/a	no	76.6% "at risk" or needed glasses
Silverstein, E.et al (2009)	Refinement of referral criteria to optimise PPV	US (Tenessee)	15749	Risk factors	1-5 yrs	yes	Suresight	Alone	46%	7%	<1%	no	max 64%
Terveen, DC,et al (2015)	Reporting experenceof photoscreening .	US (S.Dakota)	4784	Risk factors	6 mths-12yrs (mean 6.5 yrs)	yes	SPOT	Alone	n/a	11.90%	n/a	yes	n/a
